# *In cellulo* serial crystallography of alcohol oxidase crystals inside yeast cells

**DOI:** 10.1107/S2052252515022927

**Published:** 2016-01-12

**Authors:** Arjen J. Jakobi, Daniel M. Passon, Kèvin Knoops, Francesco Stellato, Mengning Liang, Thomas A. White, Thomas Seine, Marc Messerschmidt, Henry N. Chapman, Matthias Wilmanns

**Affiliations:** a Hamburg Unit c/o DESY, European Molecular Biology Laboratory (EMBL), Notkestrasse 85, 22607 Hamburg, Germany; b Structural and Computational Biology Unit, European Molecular Biology Laboratory (EMBL), Meyerhofstrasse 1, 69117 Heidelberg, Germany; c Molecular Cell Biology, Groningen Biomolecular Sciences and Biotechnology Institute, University of Groningen, 9747 AG Groningen, The Netherlands; d Center for Free-Electron Laser Science, Deutsches Elektronen Synchrotron DESY, Notkestrasse 85, 22607 Hamburg, Germany; e Linac Coherent Light Source, SLAC National Accelerator Laboratory, Menlo Park, California, USA; f Department of Physics, University of Hamburg, Luruper Chaussee 149, 22607 Hamburg, Germany; g Center for Ultrafast Imaging, Luruper Chaussee 149, 22607 Hamburg, Germany; h University Medical Center Hamburg-Eppendorf, Martinistrasse 52, 20246 Hamburg, Germany

**Keywords:** protein structure, X-ray crystallography, femtosecond studies, nanocrystals, free-electron laser

## Abstract

The possibility of using femtosecond pulses from an X-ray free-electron laser to collect diffraction data from protein crystals formed in their native cellular organelle has been explored. X-ray diffraction of submicrometre-sized alcohol oxidase crystals formed in peroxisomes within cells of genetically modified variants of the methylotrophic yeast *Hansenula polymorpha* is reported and characterized. The observations are supported by synchrotron radiation-based powder diffraction data and electron microscopy. Based on these findings, the concept of *in cellulo* serial crystallography on protein targets imported into yeast peroxisomes without the need for protein purification as a requirement for subsequent crystallization is outlined.

## Abstract 2

The application of serial femtosecond crystallography to naturally occurring peroxisomal protein crystals within yeast cells is described. The concept of utilizing peroxisomes for the production of protein nanocrystals is outlined.

## 1. Introduction   

The recent advent of X-ray free-electron lasers (XFELs) has led to rapid progress in determining three-dimensional structures from protein crystals of only several hundreds of nanometres to a few micrometres in size and with diffracting volumes up to three orders of magnitude smaller than those commonly required for data collection at conventional synchrotron sources (Chapman *et al.*, 2011 ▸; Boutet *et al.*, 2012 ▸; Redecke *et al.*, 2013 ▸). The use of XFEL radiation holds great promise to facilitate the structure determination of protein species that have so far remained recalcitrant to structural characterization by X-ray crystallography owing to difficulty in forming well ordered crystals of sufficient size. Recently developed serial femtosecond crystallography (SFX) approaches enable the collection of diffraction data prior to the emergence of radiation-induced structural disorder (Boutet *et al.*, 2012 ▸). The success of these experiments has inspired the adaption of serial data-collection strategies at synchrotron sources (serial synchrotron crystallography, SSX; Gati *et al.*, 2014 ▸; Stellato *et al.*, 2014 ▸), and has sparked considerable interest in methods to obtain, detect and optimize protein nanocrystals (Georgieva *et al.*, 2006 ▸; Gualtieri *et al.*, 2011 ▸; Kupitz *et al.*, 2014 ▸; Stevenson *et al.*, 2014 ▸).

The observation that protein crystals may form spontaneously within cells or cell organelles (Doye & Poon, 2006 ▸), however, has not been widely explored to date. Recent advancements in sample-delivery and data-collection approaches have made it possible to perform a number of proof-of-principle X-ray diffraction experiments on such samples at third-generation synchrotrons (Coulibaly *et al.*, 2007 ▸, 2009 ▸; Axford *et al.*, 2014 ▸; Gati *et al.*, 2014 ▸) and XFELs (Koopmann *et al.*, 2012 ▸; Sawaya *et al.*, 2014 ▸; Ginn *et al.*, 2015 ▸). These studies have provided an incentive for seeking strategies to systematically exploit cellular systems to produce protein crystals for SFX or SSX experiments (Koopmann *et al.*, 2012 ▸; Gallat *et al.*, 2014 ▸; Tsutsui *et al.*, 2015 ▸). In an elegant proof-of-principle experiment, Axford *et al.* (2014 ▸) determined the structure of a novel viral polyhedrin using data collected on a modern microfocus beamline from crystals of 4–5 µm in size in cryocooled insect cells mounted onto a micromesh mount. However, successful applications of SFX to determine novel protein structures *in cellulo* are still pending to date.

A primary cellular compartment in which the formation of protein crystals *in cellulo* has been reported is the peroxisome. Peroxisomes are membrane-limited organelles in eukaryotic cells with important roles in sequestered lipid metabolism and the scavenging of reactive oxygen species (Wanders & Waterham, 2006 ▸). Crystal formation of peroxisomal enzymes has been observed in a range of organisms: alcohol oxidase in yeast peroxisomes (van Dijken *et al.*, 1975 ▸; Tanaka *et al.*, 1976 ▸; Veenhuis *et al.*, 1978 ▸), uricase in rat hepatocyte peroxisomes (Hruban & Swift, 1964 ▸; Tsukada *et al.*, 1966 ▸) and catalase in plant peroxisomes (Heinze *et al.*, 2000 ▸). Here, we set out to assess the potential of SFX for solving the crystal structures of such enzymes in their native environment inside the cell.

**Figure 1:**
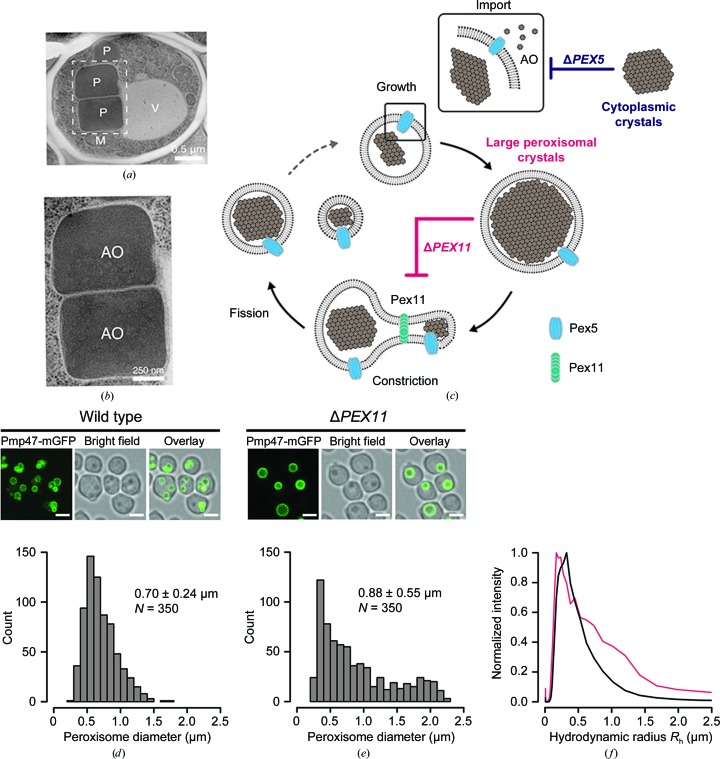
(*a*) Electron micrograph of a wt Hp cell containing crystalline alcohol oxidase (AO) in electron-dense peroxisomes (P) seen next to mitochondria (M) and a vacuole (V). The crystalline matrix is visible in the regular striated pattern observed at higher magnification (*b*). Also note the single membrane outlining the organelle and enclosing the crystal. (*c*) Schematic representation of peroxisome proliferation. Deletion of the cytosolic peroxisomal cargo receptor Pex5, which is also part of the peroxisomal translocon, prevents import of AO into the peroxisomal matrix and results in cytosolic AO crystals. (*d*, *e*) Δ*PEX11* cells display compromised fission and result in fewer (typically one) and larger peroxisomes per cell as observed by fluorescence microscopy with the peroxisomal membrane label Pmp47-mGFP. Scale bars are 2 µm in length. (*f*) Mean radius distributions from dynamic light scattering for purified fractions of wt (black) and Δ*PEX11* (red) peroxisomes.

We focused on *Hansenula polymorpha* (Hp), in which the predominant peroxisomal protein, alcohol/methanol oxidase (AO), has been observed to form submicrometre-sized crystals inside the peroxisomal matrix (Veenhuis *et al.*, 1978 ▸, 1981 ▸). AO expression in methylotrophic yeast cells is strictly regulated at the transcriptional level by methanol induction. If grown on methanol as the main carbon source, the peroxi­somal lumen is abundant in AO, which assembles into a crystalline matrix (Figs. 1 ▸ *a* and 1 ▸ *b*). Hp AO is a member of the glucose–methanol–choline oxidoreductases and catalyzes the oxidation of methanol to formaldehyde with the concomitant production of hydrogen peroxide. Unlike most other oxido­reductases, peroxisomal Hp AO in its mature form consists of eight identical subunits with a total molecular weight of approximately 600 kDa. X-ray diffraction of large Hp AO crystals grown from purified protein was found to be limited to 6 Å resolution, but no further characterization was reported (Van der Klei *et al.*, 1989 ▸). Despite historic efforts, the crystal structure of the Hp AO complex remains undetermined to date, the closest homologue of known structure being monomeric pyridoxine 4-oxidase (PDB entry 4ha6; Mugo *et al.*, 2013 ▸), with 29.4% sequence identity. Owing to the small overall dimensions of Hp peroxisomes the experimental conditions of hard X-ray FELs are well suited for diffraction experiments with this challenging target. Here, we demonstrate that SFX diffraction up to 6 Å resolution can be observed from single micrometre-sized peroxisomal AO crystallites within their native environment in intact yeast cells.

## 2. Materials and methods   

### 2.1. *H. polymorpha* growth conditions   

Yeast cultures were pre-cultured at 37°C in mineral medium (Van Dijken *et al.*, 1975 ▸) supplemented with 0.5% glucose. For AO induction, the cells were shifted into mineral medium containing 0.5% methanol as the carbon source at an OD_600_ of 0.1 and grown for 16 h at 37°C. If required, uracil and/or leucine were added to a final concentration of 30 µg ml^−1^.

### 2.2. Organelle purification   

The peroxisome organelle purification was performed with 4 l of methanol-grown cultures. The harvested cells were converted into protoplasts using Zymolase 20T (Van der Klei *et al.*, 1989 ▸) and homogenized. Peroxisomes were isolated by differential and sucrose-density centrifugation and confirmed by Western blotting with antibodies against the peroxisomal marker Pex11α (Douma *et al.*, 1985 ▸). The enriched peroxi­somal fraction was diluted with 1.5 *M* sorbitol in 5 m*M* MES pH 5.5, 0.1 m*M* EDTA, 1 m*M* KCl, 1 m*M* phenylmethyl­sulfonyl fluoride (PMSF) to a final concentration of 0.75 *M* sorbitol and 20–25% sucrose. A final buffer exchange was conducted by centrifugation at 30 000*g* for 30 min and resuspension of the organelle pellet in 1.5 *M* sorbitol, 5 m*M* MES pH 5.5, 0.1 m*M* EDTA, 1 m*M* KCl, 1 m*M* PMSF. The sample concentration was estimated to be 2 × 10^11^ particles ml^−1^ with a counting chamber and the sample was stored at 4°C.

### 2.3. Fluorescence microscopy   

Fluorescence-microscopy images of wild-type (wt) and *PEX11* gene-deficient (Δ*PEX11*) cells containing mGFP-tagged Pmp47 were captured with a confocal microscope (LSM510; Carl Zeiss) equipped with photomultiplier tubes (Hamamatsu Photonics) and the *ZEN* 2009 software. mGFP fluorescence was analyzed by excitation of the cell with a 488 nm argon-ion laser (Lasos), and emission was detected using a 500–550 nm bandpass emission filter. For quantification of peroxisome sizes, Z stacks were acquired with an interval of 0.6 µm and were analyzed using an *ImageJ* plugin (Williams *et al.*, 2015 ▸). The presented images were created by median filtering the stacks in three dimensions (2 × 2 × 2 kernel) and merging in the *z* direction by averaging.

### 2.4. Dynamic light scattering   

Serial *in situ* DLS measurements were performed with a dilution of the purified peroxisome solution with a SpectroSize 300 instrument (XtalConcepts, Germany) in a quartz cuvette with 7 µl sample volume (Hellma, Germany). The laser wavelength was set to 660 nm, 100 mW and the scattering angle of the detector placement to 90°. The dynamic viscosity parameter for sample buffer containing 1.5 *M* sorbitol was calibrated using 600 nm NIST standard polystyrene microspheres (Duke Scientific). A dynamic viscosity parameter of 2.13 cP and a refractive index of 1.33 were used for the experiment. A series of measurements (*n* = 20; 300 ms) were directly recorded after pipetting at 20°C.

### 2.5. Electron microscopy   

*H. polymorpha* wt cells were cryo-fixed in liquid ethane using the sandwich plunge-freezing method (Baba, 2008 ▸). Cells were freeze-substituted in 1% osmium tetroxide, 0.5% uranyl acetate, 5%(*v*/*v*) distilled water in acetone using the fast low-temperature dehydration and fixation method (McDonald & Webb, 2011 ▸). Cells were infiltrated overnight with Epon and polymerized for 48 h at 60°C. Sections of 200 nm in thickness were cut and overlayed with 10 nm of fiducial gold particles. Two single-axis tilt series, each containing 131 images with 1° tilt increments, were acquired on an FEI Tecnai 20 running at 200 kV using the FEI automated tomography acquisition software and a cooled slow-scan charge-coupled device camera (Ultrascan 4000; Gatan) in 2 × 2 binned mode with a final pixel size of 1.1 nm at the specimen level. The tilt series were aligned and reconstructed by the simultaneous iterative reconstruction technique (SIRT) algorithm using the *IMOD* software package (Kremer *et al.*, 1996 ▸).

### 2.6. X-ray powder diffraction   

Cell suspensions of wt, *PEX5* gene-knockout (Δ*PEX5*), Δ*PEX11* or ΔAO strains were concentrated to ∼5 × 10^9^ cells ml^−1^, transferred to a 0.1 mm glass capillary and pelleted. Capillaries were mounted on a vertically mounted goniometer of the MD3 microdiffractometer (EMBL/Bruker ASC/Arinax) at the P14 beamline at the PETRA III synchrotron, DESY, Hamburg and powder patterns were collected on a Dectris Pilatus 6M detector using an exposure time of 10 s (10^14^ photons s^−1^). Diffraction data were visual­ized and analyzed with *EVAL*15 (Schreurs *et al.*, 2010 ▸).

### 2.7. XFEL   

SFX experiments were performed on the Coherent X-ray Imaging (CXI) beamline (Boutet *et al.*, 2015 ▸) at the Linac Coherent Light Source (LCLS) using a tiled two-dimensional pixel-array detector (PAD). Data were collected from a fully hydrated stream of Δ*PEX11* and Δ*PEX5* cell suspensions at ∼5 × 10^9^ cells ml^−1^ that were fixed prior to data collection. Cells were passed through a 10 µm stainless-steel frit mounted in-line with the sample tubing to prevent clogging of the injector. The samples were supplied to the sample chamber as a gas-focused liquid jet of 5 µm diameter using a gas-dynamic virtual nozzle at a flow rate of ∼15 µl min^−1^ at 20°C. To prevent settling of cells, the suspension was agitated using a temperature-controlled anti-settling device (Lomb *et al.*, 2012 ▸). Diffraction data were recorded using a 100–300 nm FWHM beam at a photon energy of 7.925 keV (1.56 Å) with 30 fs pulse duration. The CS-PAD detector was positioned 425 mm from the sample-interaction point. Diffraction patterns from AO crystals were identified and selected using the hit-finding program *Cheetah* (Barty *et al.*, 2014 ▸). Composite powder diffraction patterns were assembled from the individual images using *Cheetah* and were visualized and analyzed using *CrystFEL* (White *et al.*, 2012 ▸).

## 3. Results   

To assess the suitability of peroxisomes as a source of nanocrystals for SFX experiments, we first purified and characterized peroxisomes from NCYC495 wild-type (wt) Hp yeast cells (Sudbery *et al.*, 1988 ▸) as well as mutant strains deficient in the *PEX11* (Krikken *et al.*, 2009 ▸) and *PEX5* genes (Salomons *et al.*, 2001 ▸). Genetic knockout of *PEX5* (Δ*PEX5*) results in a dysfunctional peroxisomal import pathway (Van der Klei *et al.*, 1991 ▸). Strains deficient in the *PEX11* gene (Δ*PEX11*) display impaired peroxisome proliferation (fission) and commonly contain only a single peroxisome per cell (Krikken *et al.*, 2009 ▸; Fig. 1 ▸ *c*).

We exploited the use of Δ*PEX11* cells to alleviate the potential problem of obtaining multiple and overlapping diffraction patterns from individual Hp cells, which despite the small beam diameter (100–300 nm) can represent a serious detriment if two or more crystals located along the beam direction interact simultaneously with a single X-ray pulse. To assess the level of sample homogeneity of wt and Δ*PEX11* cells, we quantified the peroxisome number and size inside cells in parallel by fluorescence microscopy. We fused the adenosine triphosphate transporter Pmp47 to a monomeric mutant of green fluorescent protein (Pmp47-mGFP; Figs. 1 ▸ *d* and 1 ▸ *e*) to label and measure the peroxisomal membrane outlining the AO crystals. On average, Δ*PEX11* cells contained slightly larger peroxisomes (0.88 ± 0.55 µm) than wt cells (0.70 ± 0.24 µm). The distribution of peroxisome size is more uniform in wt cells than in the Δ*PEX11* variant, with only a small proportion of peroxisomes having dimensions larger than 1 µm. The narrow size distribution, with an average diameter of approximately 700 nm, suggests that the expected experimental errors in the diffraction data resulting from sample-size inhomogeneity (Kirian *et al.*, 2010 ▸) are relatively low. These results were confirmed by dynamic light-scattering data obtained from isolated peroxisome fractions that were purified from the host cells (Fig. 1 ▸ *f*). In Δ*PEX11* cells a significant fraction of peroxisomes grew extraordinarily large, approaching a diameter of ∼2.5 µm. This size increase is explained by the preservation of the total peroxisomal volume in Δ*PEX11* cells, which accumulates in a single peroxisome. Since the diffraction signal is expected to scale with the illuminated crystal volume, we tried to further enrich Δ*PEX11* cells with large peroxisomes by fractional centrifugation on a sorbitol cushion. Gradient fractionation resulted in a marked reduction of the cell population with small peroxisomes and a moderate increase in cells with very large peroxisomes (1.1 ± 0.62 µm) relative to the untreated sample (Supplementary Fig. S1). Finally, to assess the importance of compartmentalization (in addition to size) for crystal quality, we also investigated Δ*PEX5* cells with impaired peroxisomal import. In this mutant, we expect to find AO crystals only in the cytosol (Van der Klei *et al.*, 1991 ▸), hence allowing comparison with crystals grown inside peroxisomes to yield a quantitative assessment of the effect of organelle confinement on diffraction quality.

**Figure 2:**
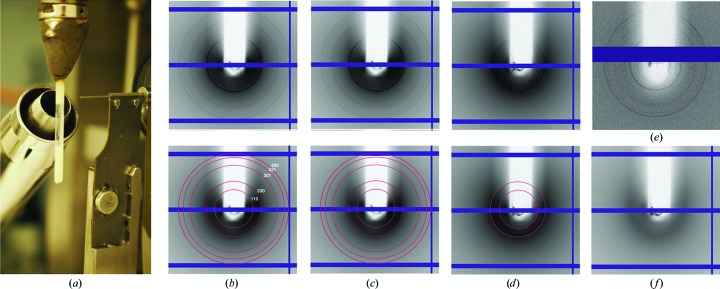
(*a*) Setup for powder diffraction experiments with cell and peroxisome suspensions on the P14 beamline at PETRA III. X-ray powder diffraction patterns are shown for (*b*) wild-type, (*c*) Δ*PEX11* and (*d*) Δ*PEX5* cells. The lower panels in (*b*), (*c*) and (*d*) indicate Debye–Scherrer rings at 161 Å (corresponding to the 110 reflection), 114 Å (200 reflection), 72 Å (301 reflection), 61 Å (321 reflection) and 57 Å (400 reflection), consistent with *d*-spacings of an *I*-centred cubic lattice with *a* = 228 Å. Reflections 211, 220 and 222 are not visible in our diffraction data. (*e*) Purified peroxisomes produced the same diffraction pattern as wt and Δ*PEX11* cells, whereas ΔAO cells with a deletion in the *AOX* gene do not produce Debye–Scherrer rings (*f*).

We induced the formation of crystalline AO by growing Hp cells on methanol-containing medium and tested the diffraction properties of concentrated cell and organelle suspensions on the EMBL/DESY beamline P14 at the PETRA III synchrotron. For all preparations we observed visible Debye–Scherrer rings extending to ∼40 Å, suggesting that all cells and purified peroxisomes investigated comprised a crystalline state of the AO matrix (Figs. 2 ▸ *a*–2 ▸ *d*). The observed *d*-spacings in the diffraction data are compatible with an *I*-centred cubic lattice and a cell edge of ∼228 Å. Of note, the 211, 220 and 222 reflections are not visible in the diffraction data. The lattice constants inferred from the powder diffraction data are supported by distances in Fourier amplitude spectra calculated from electron micrographs of crystalline AO in peroxisomes in our sample preparation (Supplementary Fig. S2). These dimensions are in agreement with previous electron diffraction data from AO crystalloids grown *in vivo* (Veenhuis *et al.*, 1981 ▸) or from purified protein (Vonck & van Bruggen, 1992 ▸). Debye–Scherrer rings are strongest for the wt and Δ*PEX11* preparations and are significantly weaker for Δ*PEX5* cells (Figs. 2 ▸ *b*, 2 ▸ *c* and 2 ▸ *d*, Supplementary Fig, S3). Purified peroxisomes from wt cells produce the same diffraction patterns as observed for whole-cell suspensions (Fig. 2 ▸ *e*), whereas reflections are absent in cell suspensions of a Δ*AO* strain (Lahtchev *et al.*, 2002 ▸) in which Hp AO expression is abrogated (Fig. 2 ▸ *f*). In summary, these experiments confirm the detection of X-ray diffraction from crystalline AO in purified peroxisomes as well as wt, Δ*PEX11* and Δ*PEX5* cells with intensities of Debye–Scherrer signals that are well beyond background levels.

Next, we tested diffraction from AO crystallites in individual cells in a coherent X-ray imaging (CXI) experiment at the Linac Coherent Light Source (LCLS). We first optimized the conditions for introducing peroxisome and yeast-cell suspensions into the X-ray beam as a thin liquid jet using a gas dynamic virtual nozzle (GDVN; DePonte *et al.*, 2008 ▸). We purified the peroxisomes on isosmotic sucrose gradients and initially stored them in high-percentage sucrose buffers as previously described (Graham, 2001 ▸). During test runs with our buffer solutions on a GDVN replica setup it became apparent that high-percentage sucrose solutions did not produce stable jets owing to rapid nozzle clogging. We therefore substituted the sucrose buffer with an isosmotic solution of 1.5 *M* sorbitol (Weast, 1986 ▸), which we could jet successfully with a GDVN nozzle. Yeast cells could be resuspended in phosphate buffer or distilled water without compromising their integrity or the diffraction properties of crystalline AO. In our experiments we used a nozzle of 50 µm inner diameter with a liquid flow rate of ∼15 µl min^−1^ to produce a stable jet of ∼5 µm diameter for the whole-cell and peroxisome suspensions. Diffraction patterns were obtained by exposing a fully hydrated stream of cells to X-ray pulses of 30 fs nominal duration and were recorded on a Cornell–SLAC pixel-array detector (CSPAD) at a frequency equal to the X-ray pulse rate (120 Hz). We collected a total of 309 496 frames for Δ*PEX11* cell suspensions and 43 056 frames for Δ*PEX5* cells. Hit-finding procedures using *Cheetah* (Barty *et al.*, 2014 ▸) characterized a total of 3404 (1.1%) patterns as single-crystal diffraction for the Δ*PEX11* cells. For the Δ*PEX5* cells, 215 (0.5%) single-crystal diffraction patterns were found. The scarcity, as well as the very low resolution and poor overall quality, of the Δ*PEX5* data did not permit any further processing.

**Figure 3:**
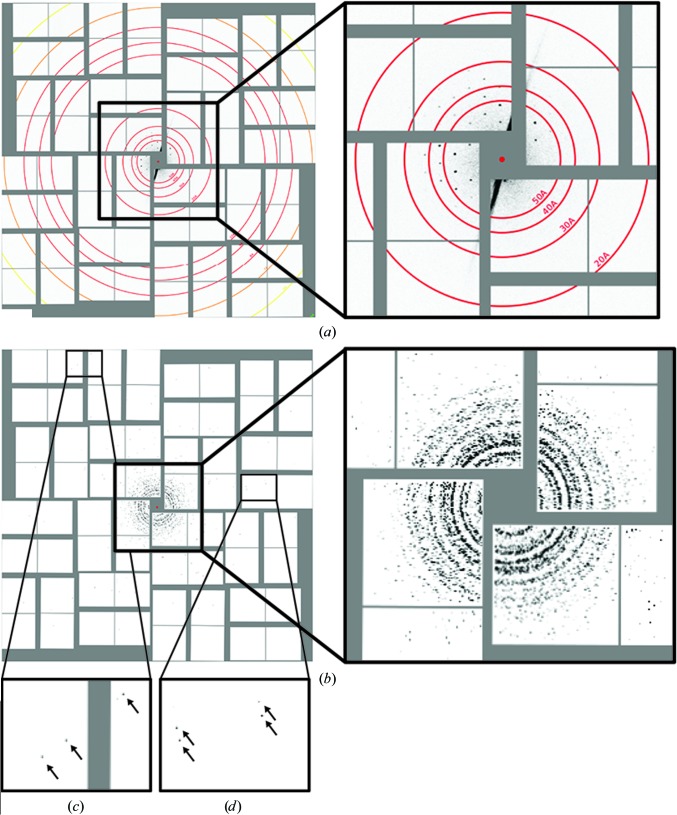
(*a*) Example SFX diffraction image of Δ*PEX11* cells displaying Bragg-sampled reflections with intensities above the background level. (*b*) Composite XRPD patterns assembled from individual diffraction images show that most crystallites diffract to approximately 30 Å resolution, with several crystals displaying diffraction out to the detector edge (6 Å) and corners (5.6 Å) as indicated by arrows in insets (*c*) and (*d*).

Inspection of individual Δ*PEX11* images revealed well resolved Bragg-sampled diffraction patterns, indicating single-crystal diffraction (Fig. 3 ▸ *a*). Owing to the low resolution, however, single images contained too few Bragg peaks to be indexed robustly by *CrystFEL* or *cctbx.xfel* (White *et al.*, 2012 ▸; Sauter *et al.*, 2013 ▸). For overall comparison of the SFX data and the X-ray powder diffraction (XRPD) patterns collected at the PETRA III synchrotron, we therefore generated composite powder patterns by summing all individual SFX diffraction images that contain Bragg peaks. The limited number of diffracting crystallites led to incompletely sampled but discernible Debye–Scherrer rings in the composite powder patterns (Fig. 3 ▸ *b*). While the majority of diffraction patterns are restricted to approximately 30 Å, we occasionally observed diffraction up to the detector edge at 6 Å (Figs. 3 ▸ *c* and 3 ▸ *d*), thus suggesting that the highest attainable resolution was possibly limited by the experimental geometry. From the size distribution of the Δ*PEX11* peroxisomes (Fig. 1 ▸ *e*) and the lattice constants derived from the diffraction data, we estimate that the crystals used for the SFX experiments consisted of approximately 10 000 (0.5 µm; 0.125 µm^3^) to 670 000 (2 µm; 8 µm^3^) unit cells. Assuming a beam cross-section ranging from 0.008 to 0.07 µm^2^ leads to an estimation of 330 to 12 000 unit cells contained in the illuminated crystal volume at a centred beam crossing. In view of the moderate hit rate and low resolution, we consider it unlikely that diffraction from the smallest crystals is observed. Assuming one or two molecules per asymmetric unit as deduced from electron microscopy (Vonck & van Bruggen, 1992 ▸), we estimate the solvent content in the putative *I*-centred cubic lattices as 63 or 75%. This figure is significantly larger than for structures solved from similarly small crystals (Chapman *et al.*, 2011 ▸; Sawaya *et al.*, 2014 ▸; Ginn *et al.*, 2015 ▸) and could represent one reason why high-resolution diffraction of AO crystals has been impossible to obtain to date.

## 4. Discussion   

We demonstrate that SFX is able to detect *in cellulo* distinct diffraction properties of a large protein complex, octameric Hp AO, crystallized in its native cellular compartment. Hp AO has not been amenable to high-resolution structure determination to date, despite substantial efforts both by electron microscopy and X-ray crystallography (Veenhuis *et al.*, 1981 ▸; Van der Klei *et al.*, 1989 ▸; Vonck & van Bruggen, 1990 ▸), and therefore presents a challenging protein target for structure determination. Assuming that the previously grown Hp AO crystals (Van der Klei *et al.*, 1989 ▸) were at least 100 µm in size (no details were reported in Van der Klei *et al.*, 1989 ▸), the *in vivo* grown crystallites used here contained 1/10^6^ of the number of unit cells or less given an estimated size of approximately 1 µm or less. Hence, we believe that it has been a significant milestone to achieve a comparable resolution limit of 6 Å for such a challenging sample using SFX.

*In cellulo* crystallization in peroxisomes, as we have pre­sented here, in principle allows the use of either isolated peroxisomes or entire yeast cells with intracellular peroxisomes. The latter are intuitively expected to increase the background scatter substantially as a result of additional scattering components from nonperoxisomal cell material including membranes and cell wall. Perhaps surprisingly, therefore, our powder diffraction data obtained with isolated peroxisomes and whole yeast cells suggest that the scattering from other cellular components does not detrimentally affect the data quality. This is in line with findings reported by others (Axford *et al.*, 2014 ▸; Sawaya *et al.*, 2014 ▸). On the other hand, the increased mechanical stability of entire yeast cells may present an advantage in view of the experimental conditions required for sample preparation and sample delivery for SFX data acquisition. We made use of a genetically modified Hp variant, Δ*PEX11*, which impairs peroxisome fission to avoid the presence of overlapping diffraction patterns from crystalline material in different peroxisomes that are simultaneously interacting with the X-ray beam. The use of Δ*PEX11* cells has the additional advantage of allowing the optimization of growth conditions such that an overwhelming proportion of the yeast-cell cytoplasm is filled with crystalline material from a single peroxisome, thus increasing the diffraction signal.

Another variant, leading to a cell phenotype in which AO crystals form in the cytosol owing to dysfunctional Pex5-dependent cargo translocation, did not produce any useful diffraction data. A plausible explanation is the loss of favourable conditions for Hp AO crystallization outside the peroxisomal lumen. Compartmentalization and directed import are likely to allow a substantially higher local protein concentration than can be reached by freely diffusing AO in the cytosol, and in addition present a natural ‘purification’ step separating the crystallization process from the numerous contaminating proteins present in the cytosol. This is in agreement with previous data demonstrating that spatial confinement lowers the solubility threshold of protein solutions and positively affects their crystallization tendency (Tanaka *et al.*, 2004 ▸).

With the aim of identifying experimental conditions that sufficiently improve the diffraction of *in vivo*-grown AO crystals to solve the Hp AO structure, we are working towards a systematic characterization of variations in experimental parameters such as modulation of growth conditions, improved yeast strains, diagnostic tools for crystal identification and characterization *in cellulo*, and different forms of sample delivery.

Our long-term goal is to exploit the amenability of Hp and other yeast strains to genetic manipulation for the structural determination of various protein targets. Proteins tagged with a peroxisomal translocation signal (PTS) tripeptide at the carboxyl-terminus are translocated from the cytosol into the peroxisomal matrix (Purdue & Lazarow, 1994 ▸; Rachubinski & Subramani, 1995 ▸). Heterologous expression of target proteins with such a PTS signal under the strong *AOX* promoter in ΔAO strains may allow the protein of interest to be sorted and focally concentrated into peroxisomes for crystal formation. In principle, adjusting growth conditions provides the possibility of controlling the rate of protein expression, subcellular sorting or the rate of peroxisomal import and thereby influence the extent of supersaturation and the rate of crystal growth *in vivo*. The lessons learned from the present study will help to address the important experimental challenges lying ahead for intracellular crystal formation and its exploitation for the structure solution of biological macromolecules. Our results provide a promising starting point to foster efforts aimed at developing *in cellulo* crystallization into a useful alternative to other crystallization strategies.

## Supplementary Material

Supporting Information.. DOI: 10.1107/S2052252515022927/mf5012sup1.pdf
