# Supplementary material for: In cellulo serial crystallography of alcohol oxidase crystals inside yeast cells
Source: IUCrJ. 2016 Jan 12;3(Pt 2):88–95. doi: 10.1107/S2052252515022927 (PMC4775156; doi:10.1107/S2052252515022927)
Supplement: Supplementary file 1 [file m-03-00088-sup1.pdf]

IUCrJ

Volume 3 (2016)

Supporting information for article:

In cellulo serial crystallography of alcohol oxidase crystals inside yeast cells

Arjen J. Jakobi, Daniel M. Passon, Kèvin Knoops, Francesco Stellato, Mengning Liang, Thomas A. White, Thomas Seine, Marc Messerschmidt, Henry Chapman and Matthias Wilmanns

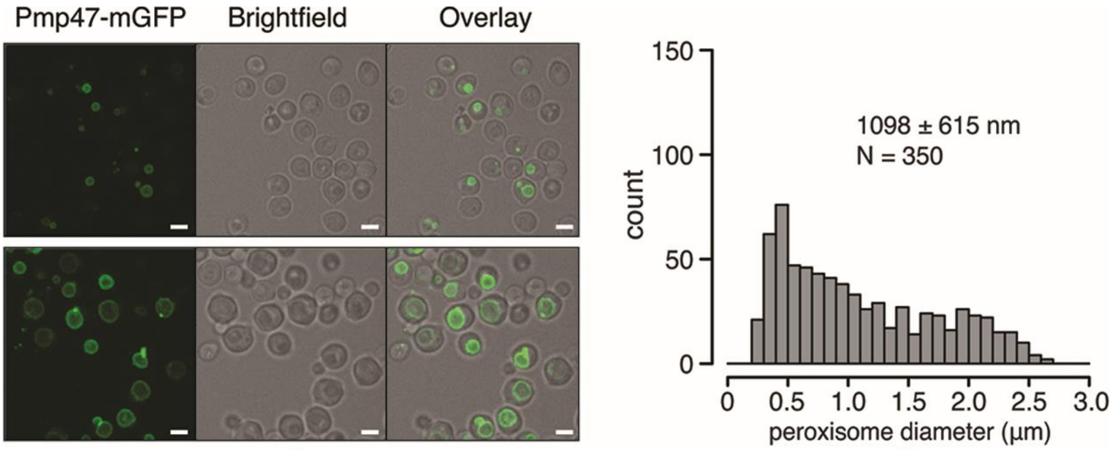

(*a*) (*b*)

Figure S1 (a) Brightfield and fluorescence images for total (top) and enriched (bottom) fractions of Δ*PEX11* cell suspensions. Scale bars represent 2 µm. (b) Size distribution of Pmp47-mGFP labeled Δ*PEX11* peroxisomes from enriched fractions of Δ*PEX11* cells show an increase in the fraction of very large peroxisomes (cf. Figure 1d–e).

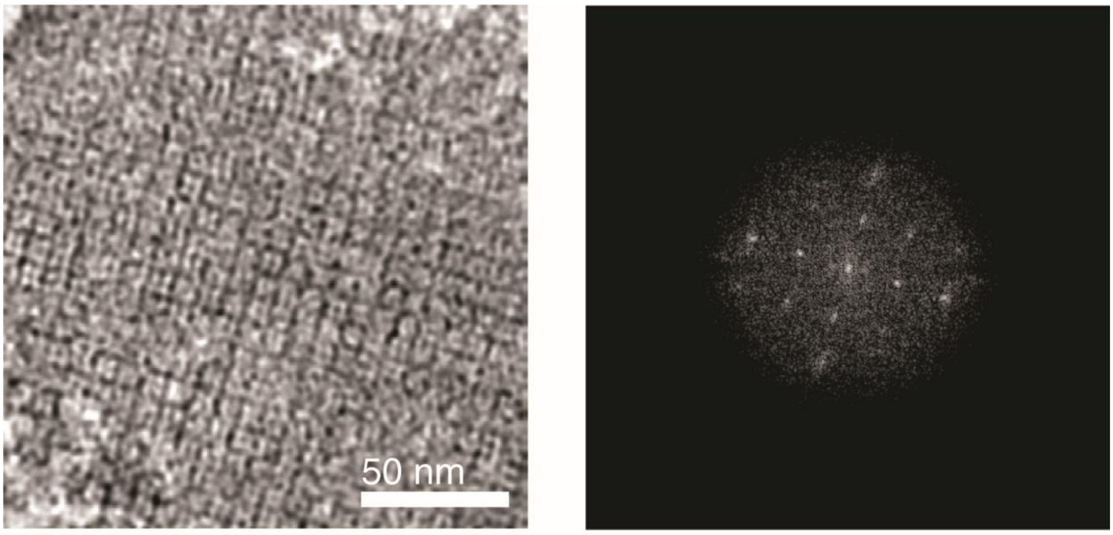

(*a*) (*b*)

Figure S2 (a) Representative digital electron tomogram slice of freeze-substituted AO crystallites in *Hp* peroxisomes with tetragonal arrangement of AO octamers viewed along [001]. (b) Fourier amplitude spectrum of (a) reflecting the discrete cubic lattice of AO in *Hp* peroxisomes.

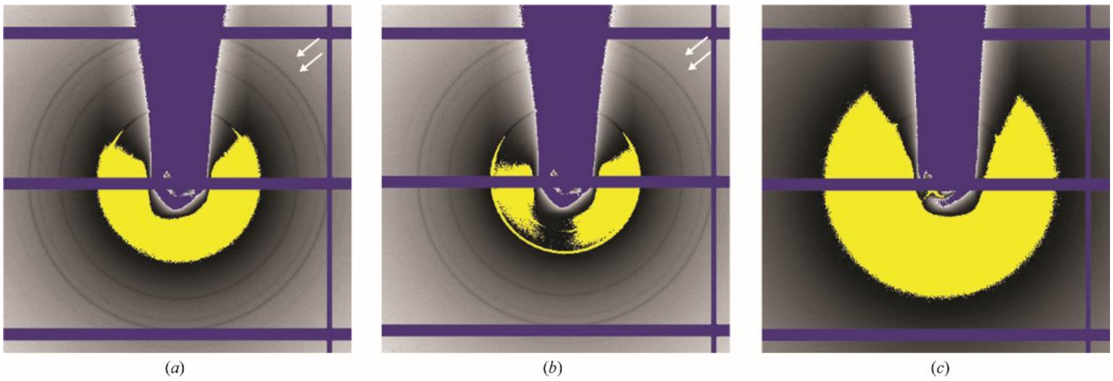

Figure S3 X-ray powder diffraction patterns of (a) *wt*, (b) Δ*PEX11* and (c) Δ*PEX5* cells at higher threshold level than shown in Figure 2 (b–d) reveal additional, higher resolution Debye-Scherrer rings for *wt* and Δ*PEX11* cells (arrows).
